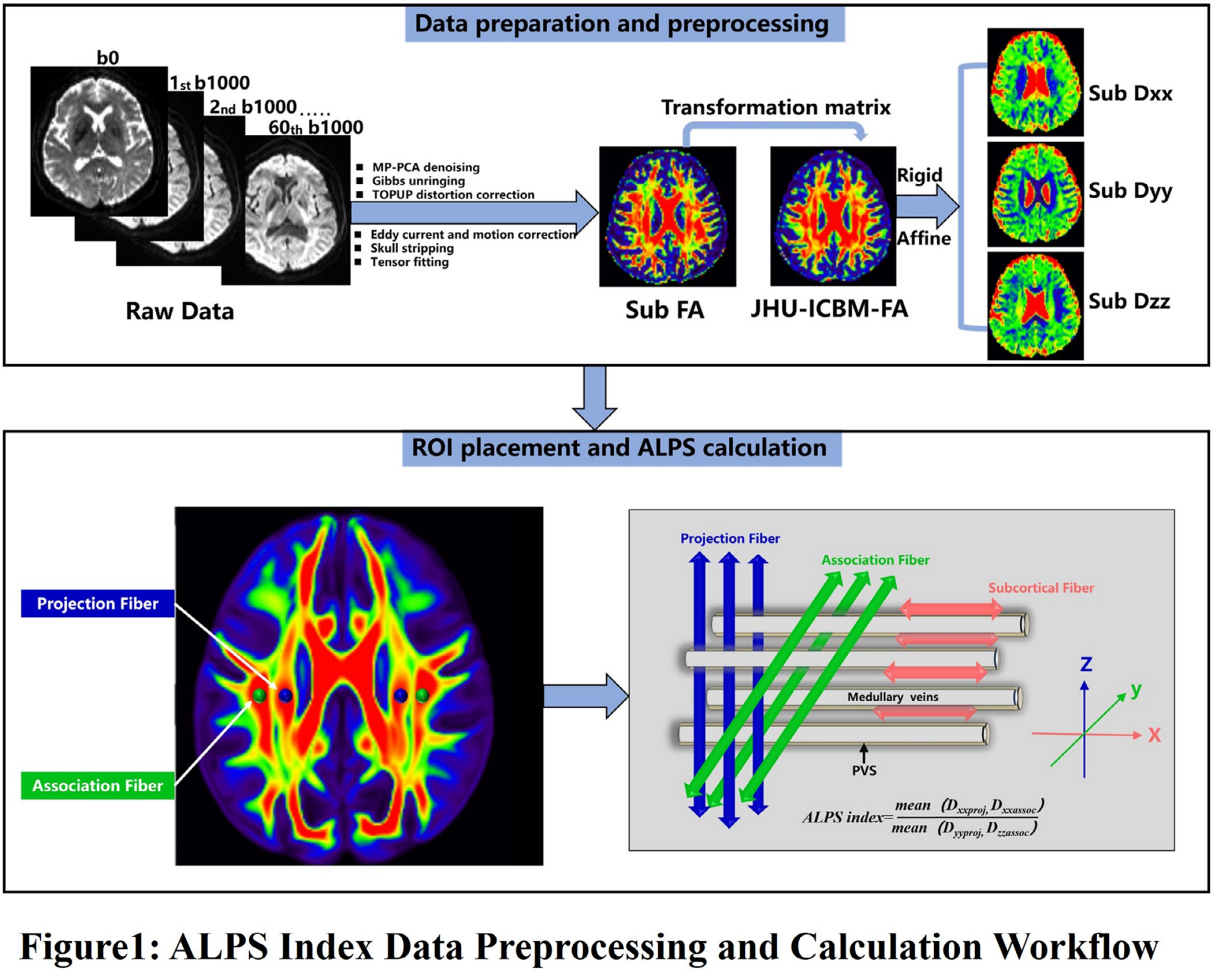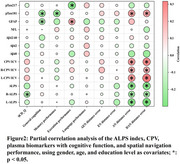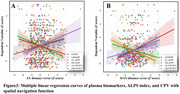# Glymphatic Dysfunction Correlate with Spatial Navigation Deficits in Subjective Cognitive Decline: Insights from 5.0T MRI and Plasma Biomarkers Analysis

**DOI:** 10.1002/alz70856_098139

**Published:** 2025-12-24

**Authors:** Futao Chen, Bing Zhang, Qian Chen, Xiang Fan

**Affiliations:** ^1^ Department of Radiology, Nanjing Drum Tower Hospital, Affiliated Hospital of Medical School, Nanjing University, Nanjing, Jiangsu, China; ^2^ Nanjing Drum Tower Hospital, Affiliated Hospital of Medical School, Nanjing University, Nanjing, Jiangsu, China; ^3^ Peking University Shenzhen Hospital, Shenzhen, Guangdong, China

## Abstract

**Background:**

Glymphatic system dysfunction is implicated in cognitive impairments in Alzheimer's Disease (AD), particularly in early symptomatic stages like subjective cognitive decline (SCD). The link between glymphatic function and spatial navigation, a key early symptom, is not well‐understood. This study evaluates glymphatic function in SCD individuals through diffusion tensor image analysis of the perivascular space (DTI‐ALPS) and choroid plexus volume (CPV) measurements, and explores their relationship with spatial navigation abilities using ultra‐high‐field 5.0T MRI.

**Method:**

From May 2023 to January 2024, 62 SCD patients and 62 matched controls underwent 5.0T MRI, spatial navigation tests, cognitive evaluations, and plasma biomarker analyses. We used the ALPS index from DTI data and CPV from 3DT1WI to assess glymphatic function, analyzing these data alongside spatial and cognitive metrics using MANOVA, partial correlation, multiple regression, and mediation analysis.

**Result:**

The SCD group exhibited significant spatial navigation deficits and glymphatic dysfunction, characterized by lower ALPS indices and higher CPV compared to controls. Glymphatic measures correlated with navigation errors, with regression analysis highlighting predictive relationships between DTI‐ALPS, CPV, and navigation performance. Mediation analysis confirmed the ALPS index's role in linking CPV to spatial navigation deficits.

**Conclusion:**

Glymphatic dysfunction correlates with impaired spatial navigation in early SCD, suggesting its potential as a physiological marker for early AD diagnosis.